# Editorial: New strategies for the treatment of diseases caused by trypanosomatid parasites

**DOI:** 10.3389/fcimb.2023.1232709

**Published:** 2023-06-12

**Authors:** Flávia de Oliveira Cardoso, Fernando Almeida-Souza, Ana Cláudia Maretti-Mira, Ana Lúcia Abreu-Silva

**Affiliations:** ^1^ Protozoology Laboratory, Oswaldo Cruz Institute, Oswaldo Cruz Foundation, Rio de Janeiro, Brazil; ^2^ Post-Graduate in Animal Sciences, State University of Maranhão, São Luís, Brazil; ^3^ Research Center for Liver Diseases, Division of Gastrointestinal and Liver Diseases, Department of Medicine, Keck School of Medicine, University of Southern California, Los Angeles, CA, United States

**Keywords:** trypanosomatids, Chagas disease, leishmaniasis, parasite, treatment, diagnosis

Neglected tropical diseases (NTDs) affect millions of people worldwide, mostly in low-income tropical countries. Among them, we highlight Chagas disease (CD) and Leishmaniasis caused by *Trypanosoma cruzi* and *Leishmania* spp., respectively, as especially important in Latin America. These diseases still represent a significant public health challenge due to limited access to health care, poverty, and inadequate treatment. Here, we discuss cutting-edge research articles that bring expressive advancement for CD and Leishmaniasis control. From the application of metagenomic for accurate diagnosis, the exploration of natural compounds, and innovative combinations of existing drugs to nanotechnology advancements to treatment, these studies offer a comprehensive overview of the current state of research in this field.

Three articles explored new options for CD and Leishmaniasis treatment. The current treatment options for CD, such as benznidazole (Bz) and nifurtimox, often face limitations in terms of efficacy and safety. Barbosa et al. explored alternative treatment approaches by combining the existing drugs Bz and amiodarone (AMD) as a more effective treatment strategy for CD. The synergism between Bz and AMD was demonstrated *in vitro* compared to monotherapy and resulted in parasite growth reduction, cardiac cell function improvement, and decreased toxicity. Bz-AMD treatment also showed promising results in restoring the cytoskeleton architecture and gap junction integrity in *T. cruzi*-infected cardiac cells. These findings provide valuable insights into the development of alternative therapeutic approaches for CD, highlighting the importance of exploring drug repurposing and combination strategies in the fight against NTDs. Additional preclinical and clinical studies are necessary to evaluate Bz-AMD therapy safety, efficacy, and pharmacokinetics in CD patients. Similarly, the existing Leishmaniasis treatment options also face challenges, such as drug resistance. Silva-Silva et al. investigated novel antileishmanial compounds, with monomethylsulochrin showing the most promising results. This natural compound showed potent activity against *Leishmania amazonensis*, *in vitro* and *in vivo*, by inducing *Leishmania* mitochondrial dysfunction. Monomethylsulochrin showed superior selectivity against intracellular *Leishmania* forms compared to amphotericin B. These findings unlock new possibilities for developing less toxic and more effective targeted therapies for Leishmaniasis.

In addition to the search for new and more efficient medication, the innovation of delivery methods for the current drugs can also reinvigorate the therapy strategies. Scariot et al. reviewed the use of nanocarriers in the management of both Leishmaniasis and CD. Nanotechnology offers ground-breaking solutions for drug delivery, diagnostics, and disease monitoring due to its unique properties at the nanoscale level. By encapsulating drugs within nanoparticles, researchers sought to improve drug solubility, protect them from degradation, and enhance their specific accumulation in infected tissues while minimizing adverse effects in healthy tissues. Furthermore, the authors explored the potential of nanosystems for vaccine delivery that could improve immune responses and provide long-lasting protection against these parasitic diseases.

In addition to the proper therapy, effective disease management requires an accurate and timely diagnosis. Two new methods were efficient for trypanosomiasis detection. Scariot et al. proposed nanotechnology-based diagnostic tools for *Leishmania* spp. and *T. cruzi* detection using nanosensors and nanobiosensors, which identify parasite-specific biomarkers and offer rapid and sensitive diagnostic capabilities. Zhang et al. successfully applied the metagenomic next-generation sequencing (mNGS) for early Visceral Leishmaniasis (VL) diagnosis. VL is the most severe form of Leishmaniasis, affects vital organs, and can be fatal if left untreated. The conventional diagnostic methods for VL have limitations regarding their sensitivity, specificity, and invasiveness. mNGS is a powerful high-throughput technique able to sequence and identify nucleic acids from a wide range of pathogens in clinical samples. Through this technology, the authors identified and characterized the *Leishmania* strain in the patient peripheral blood before detecting the parasite in the bone marrow smear, the gold-standard method for VL diagnosis. The use of mNGS offers several advantages, including the detection of parasites at low levels, identification of mixed infections, and access to the genetic diversity of the parasite population. Nevertheless, more sensitive technology for early and precise diagnosis depends on a more detailed molecular characterization of parasite species and their strains. Wen et al. showed the importance of the gene PAG3 in differentiating *Trypanosoma brucei* from other trypanosome strains. This article brings a valuable tool for differential diagnosis. *T. brucei* causes lethal African trypanosomiasis in humans and cattle. However, *T. brucei* has a nuclear genome significantly similar to two other trypanosomes, *Trypanosoma evansi* and *Trypanosoma equiperdum*. Although *T. evansi* and *T. equiperdum* do not infect humans, they cause economically significant diseases infecting camels, horses, and other domestic animals. Altogether, these findings facilitate early disease detection, enabling timely interventions and improved patient outcomes.

All five articles presented in this editorial collectively draw attention to the ongoing efforts and advancements in the battle against NTDs. These findings offer hope for better treatment outcomes and enhance public health interventions in regions heavily burdened by these diseases. With continued research and collaborative efforts, we can strive to overcome the challenges posed by NTDs and improve the lives of millions of individuals worldwide.

## Author contributions

All authors listed have made a substantial, direct, and intellectual contribution to the work and approved it for publication.

